# A Kidney-Microbiome Short- and Medium-Chain Fatty Acid Loop Mediated by OAT1: Implications for the Remote Sensing and Signaling Theory

**DOI:** 10.3390/ijms27114942

**Published:** 2026-05-29

**Authors:** Vladimir S. Ermakov, Kian Falah, Sanjay K. Nigam

**Affiliations:** 1 Department of Biology, University of California San Diego, La Jolla, CA 92093, USA; 2 Department of Pediatrics, University of California San Diego, La Jolla, CA 92093, USA; 3 Department of Medicine (Nephrology), University of California San Diego, La Jolla, CA 92093, USA

**Keywords:** Drug transporter, ADME, pharmacokinetics, Remote Sensing and Signaling Hypothesis, uremia, proximal tubule secretion, p-cresol sulfate, aryl hydrocarbon receptor

## Abstract

Short-chain fatty acids (SCFAs) and medium-chain fatty acids (MCFAs) include small organic anions derived from the gut microbiome that interact with organic anion transporters of the SLC22 family, many of which are expressed in the kidney proximal tubule. According to the Remote Sensing and Signaling Theory (RSST), crosstalk between organs (e.g., gut–liver–kidney axis, gut–brain axis) and the gut microbiome is mediated by metabolites and signaling molecules transported by multi-specific “drug” transporters. The renal drug transporter OAT1 (SLC22A6) is also a major transporter of gut-microbiome products and uremic toxins (e.g., indoxyl sulfate); it has been shown to act as part of a regulatory feedback loop involving the gut microbiome. SCFAs, especially propionate and butyrate, have been shown to play a central role in the transcriptional regulation of OAT1 through HDAC inhibition. By fecal metagenomics analyses of *Oat1* knockout mice, we now find that propionate synthesis is among the most altered pathways in the gut microbiome. In contrast, these pathways were only minimally altered in the Oat3 (Slc22a8) knockout. Metabolomics analyses indicate that serum propionate derivatives (e.g., propionyl glycine) and 3-hydroxybutyrate are dependent on OAT1 in the knockout mice and in humans treated with probenecid, an OAT1 inhibitor. The gut microbiome of the *Oat1* knockout mice also exhibited greater fatty acid synthesis, which generates odd-chain-length fatty acids (e.g. heptanoate) when propionate is available. Overall, the data, especially when considered in light of in vitro experiments of others, indicates the in vivo existence of a feedback loop connecting gut-microbiome-derived SCFAs and MCFAs to kidney proximal tubule uptake via OAT1. This bidirectional feedback loop in turn regulates OAT1 expression through HDAC inhibition. The feedback loop is clearly consistent with the Remote Sensing and Signaling Theory—in particular, the centrality of multi-specific “drug” transporters in organ crosstalk and host–microbiome interactions via small molecules with “high information content.” The key role of OAT1 function in maintaining tubular secretion in CKD supports the importance of this RSST loop in renal pathophysiology. Modulating this RSST loop could have therapeutic value in chronic kidney disease and other contexts.

## 1. Introduction

Recent evidence indicates that the multi-specific “drug” transporter, OAT1 (SLC22A6), plays a central role in the interactions of the gut microbiome with the kidney [1,2]. OAT1 is the major transporter of small, organic anions in the proximal tubule of the kidney [3,4], and it has been implicated by multiple groups in Remote Sensing and Signaling along the gut–liver–kidney axis [1,5,6,7,8,9,10,11,12,13,14].

For example, OAT1 is a major transporter of tryptophan derivatives from the gut microbiome which bind ligand-activated transcription factors (e.g. Aryl Hydrocarbon Receptor or AHR) and G-protein-coupled receptors (GPCRs) [15,16]. OAT1 is also necessary for urate excretion by the kidney, and the microbiome in the *Oat1* KO mouse compensates for decreased urate excretion by the kidney by decreasing its own urate production [1]. Furthermore, gut microbiome metabolites like indoxyl-sulfate work together with signaling pathways involving AHR, epidermal growth factor (EGF), and a micro-RNA to regulate OAT1-mediated excretion of indoxyl sulfate by the kidney [5].

These examples of feedback loops involving *Oat1* have been viewed as supporting the Remote Sensing and Signaling Theory (RSST), which emphasizes the central role of multispecific “drug” transporters in homeostasis via the regulation of small molecule crosstalk between organs and organisms [10,17,18]. Another proposed *Oat1*-dependent example of remote sensing and signaling between the gut microbiome and the kidney is short-chain fatty acids (SCFAs). Gut-microbiome-derived metabolites such as propionate and butyrate regulate the expression of OAT1 through changes in histone acetylation, including inhibition of histone deacetylases (HDACs) [19]. Various OATs (e.g., OAT1, OAT6) are known to interact with short- and medium-chain fatty acids (MCFAs), as well as their derivatives [20,21]. Thus, current data raises the possibility of an *in vivo* feedback loop involved in proximal tubule remote communication with the microbiome via short-chain fatty acids [19].

The aforementioned data suggests that OAT1 expression is regulated by SCFAs derived from the gut microbiome. Furthermore, OATs are known to directly interact with signaling fatty acids in in vitro transport assays [20,21,22]. However, it is crucial to determine whether this is true in vivo. Here, we analyze this question in-depth. We find that the *Oat1* KO mouse microbiome has major alterations in synthesis pathways involving propionate and 3-hydroxybutyrate, small molecules believed to be important in signaling in the kidney and other organs. Medium-chain fatty acid synthesis is upregulated in the *Oat1* KO microbiome as well. The results support an in vivo homeostatic role for SCFA and MCFA microbiome pathways in response to the loss of renal OAT1 function. Moreover, reanalysis of our metabolomics data from the *Oat1* KO mouse and humans treated with an OAT1 inhibitor revealed altered propionate derivatives. When taken together with recent in vitro data published by others [19], our data provides strong in vivo support for an OAT1-dependent RSST loop regulating SCFAs and MCFAs that requires mutual interactions between the kidney and gut microbiome. These in vivo results indicate several levels at which SCFA and MCFA signaling can be therapeutically regulated in chronic kidney disease (CKD) and other medical contexts.

## 2. Results

### 2.1. The Microbiome of Oat1 KO Mice Has Marked Increases in Propionate-Producing Bacteria

A number of lines of evidence indicate that histone modifications (e.g., acetylation, methylation) play a key role in the epigenetic regulation and expression of OAT1 [19,23,24]. This is also seen in vivo during kidney development, where the loss of HDACs in knockout mice affects OAT1 expression [25]. In addition, in our previously published data on epigenetic regulation of transporters and enzymes [23], we noted changes in histone modifications in chromatin surrounding the *Oat1* locus in adults compared to embryonic kidney within the timeframe of known marked changes in OAT1 expression and transport function [26,27]. Furthermore, it has recently been demonstrated that OAT-transported short-chain fatty acids act as HDAC modulators and regulate OAT1 expression in vitro in microphysiological systems modeling the kidney [19]. Thus, there is a growing body of evidence, both in vivo and in vitro, indicating a central role for histone acetylation in the regulation of OAT1 expression. However, the specific role of the gut microbiome, which is the source of the small molecules regulating OAT1 expression, has not been assessed. We analyzed this in-depth in the *Oat1* knockout mice.

**Figure 1:**
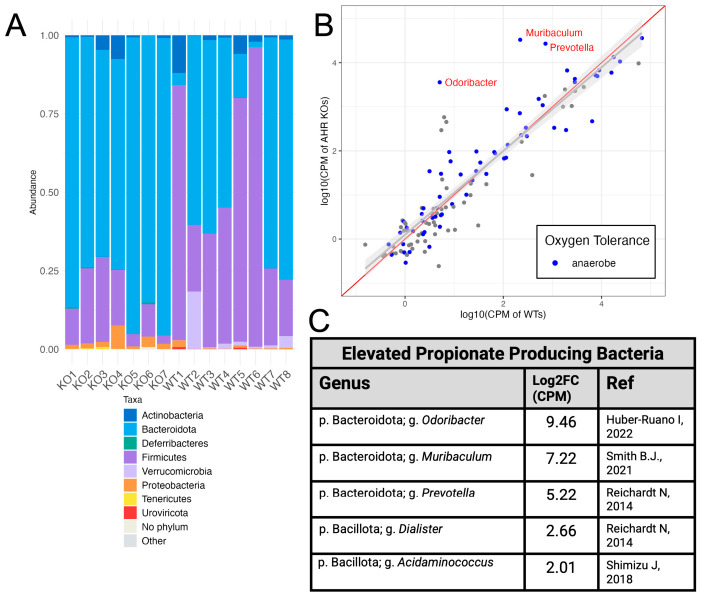
Compositional changes in the *Oat1* KO show increases in propionate-producing taxa. (**A**) Taxa bar chart showing the relative abundance of phyla in the *Oat1* KO and WT microbiomes. *Oat1* KOs have a clear expansion of the phyla *Bacteriodota* compared to the wildtype, which are propionate producing. The wildtype mouse microbiomes have a greater abundance of *Firmicutes*, which are commonly butyrate producers. (**B**) Log-transformed Counts Per Million (CPM) of reads assigned to genera in the *Oat1* KO and WT microbiomes. Blue-highlighted points represent genera classified as obligate anaerobes. A 95% confidence area is shaded in gray. Anaerobic genera appear to favor the *Oat1* KO. (**C**) Table of propionate-producing genera enriched in the *Oat1* KO with log2FC (CPM Oat1KO/CPM WT) [31,32,33,34].

Shotgun metagenomics was performed on *Oat1* KO (*n* = 7) and wildtype (*n* = 8) mice to understand the taxonomic and functional differences between the two groups. Relative compositions of phyla present in the *Oat1* KO and WT mice were measured and then plotted on a taxa plot (Figure 1A). When comparing the knockouts and wildtypes, we found a clear reduction in *Firmicutes* phyla in the *Oat1* KO compared to the wildtype. *Firmicutes* are generally agreed to constitute the majority of phyla in the mouse gut microbiome (>50%) [28], which we observe in our WT mice. However, the *Oat1* KO *Firmicutes* appear to constitute <25% of the measured phyla. Conversely, we observed a relative expansion of *Bacteroidota* in the *Oat1* KO, which make up >70% of the phyla measured in the *Oat1* KO microbiomes. Interestingly, previous studies have shown that *Firmicutes* phyla are generally butyrate-producing, whereas *Bacteroidota* are generally propionate-producing [29,30]. Since OATs transport a range of SCFAs and their derivatives—and because the loss of other renal organic anion transport (e.g., urate) in the *Oat1* KO results in reciprocal changes in microbial pathways to compensate for the loss of renal transport—we analyzed taxonomical changes in the *Oat1* KO mouse with respect to SCFA production.

Short-chain fatty acids are generally produced by anerobic bacteria in the gut. We examined the genera present in both groups and compared their abundance using log transformed Counts Per Million (CPM), highlighting the genera classified as obligate anaerobes. There were several anaerobic genera favored in the *Oat1* KO. Some of the most highly elevated anaerobic genera in the *Oat1* KO included: *Odoribacter* (9.46 Log2FC), *Muribaculum* (7.22 Log2FC), and *Prevotella* (5.22 Log2FC) (Figure 1B,C). These are known propionate-producers. Additionally, several other propionate-producing genera appear to be more-abundant in the *Oat1* KO, including: *Dialister* and *Acidaminococcus* (Figure 1C). The *Oat1* KO microbiomes appear to strongly favor anaerobic genera, in particular those that produce propionate.

### 2.2. Propionate Synthesis Is Markedly Elevated in the Gut Microbiomes of Oat1KO Mouse as Revealed by Whole Genome Sequencing

The taxonomic changes between the *Oat1* KO and WT microbiomes prompted us to investigate whether there was functional enrichment of SCFA synthesis in the *Oat1* KO. Metagenomic sequencing reads were used to assess functional changes in the knockout and WT microbiomes. The reads were assembled into protein coding sequences (CDSs) and annotated using the Kyoto Encyclopedia of Genes and Genomes (KEGG) database. A differential abundance analysis of bacterial genes was performed using DESEQ2, allowing us to assess statistically significant changes in gene abundance. In line with our previous findings [1], we found urate production to be decreased in the *Oat1* KO microbiomes.

**Figure 2:**
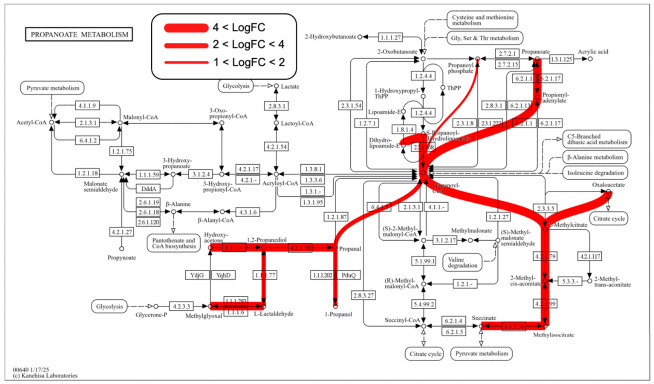
Pathway diagram of significantly elevated bacterial enzymes involved in propionate synthesis in the *Oat1* KO microbiome. The KEGG pathway: ko00640 (Propanoate metabolism) was plotted with the results of the DESEQ2 analysis. Red highlights indicate one or more significantly elevated enzymes (adj. *p*-value < 0.05) in the *Oat1* KO gut bacteria for an enzymatic step. Thickness of the highlight indicates Log2 fold-change. The conversion of succinate to propionate appears to be the highly enriched, with each step of the pathway greatly enriched (Log2FC > 4, adj. *p*-value < 0.05). Minor propionate synthesis pathways, via 1,2-propanediol are also significantly enriched.

We mapped significantly elevated genes onto the KEGG pathway: ko00640 (Propanoate metabolism) and found enrichment of two propionate synthesis pathways (Figure 2) in the *Oat1* KO microbiome. The most elevated of the two pathways (adj. *p*-value < 0.05, average log2FC > 4) (Table 1) was propionate synthesis via succinate metabolism, which has previously been shown to be the major propionate-synthesizing pathway in the gut [31]. Propionate synthesis via 1,2-propanediol was also found to be significantly elevated in the *Oat1* KO, but to a lesser extent than the succinate-dependent pathway.

Remarkably, there was a significant elevation of every step in these two synthesis pathways (Figure 2). In our experience of analyzing gut microbiomes of multi-specific transporter knockout mice, this degree of elevation (Log2FC > 4) across all steps in a pathway that produces a key signaling molecule is unusual. Taken together with the taxonomical changes mentioned earlier, this provides strong evidence that the *Oat1* KO microbiomes have marked elevations in propionate synthesis.

### 2.3. Whole Genome Sequencing of the Oat1 KO Microbiome Reveal Elevations in Bacterial Pathways Leading to the Production of 3-Hydroxybutyrate, a Known Oat1 Substrate That Is Also Elevated in the Oat1 KO Plasma

**Figure 3:**
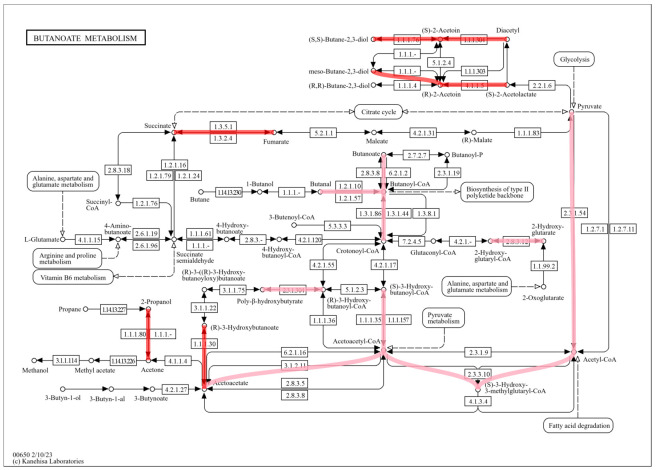
Pathway diagram of C4 SCFA producing pathways. Red-highlighted enzymatic steps indicate a significant enrichment (adj. *p*-value < 0.05) in the *Oat1 KO* microbiomes. The enzyme 3-hydroxybutyrate dehydrogenase [EC: 1.1.1.30] catalyzing 3-hydroxybutyrate interconversion from acetoacetate is significantly elevated in the *Oat1* KO microbiomes. Pink-highlighted pathways indicate elevated in the knockout (Log2FC > 0), but did not meet the significance threshold. Butyrate production does not appear significantly elevated in the *Oat1* KO microbiomes, which is consistent with the decrease in butyrate-producing bacteria (e.g., *Firmicutes* phyla) in the *Oat1* KO microbiomes (Figure 1A).

Given the marked elevations of propionate synthesis across all enzymatic steps in the microbial pathway of the *Oat1* KO, we sought to understand if the earlier taxonomic predictions held true for butyrate synthesis. We mapped bacterial genes enriched in the *Oat1* KO to the KEGG pathway: ko00650 (Butanoate metabolism) (Figure 3).

While several steps in butyrate synthesis were detected in the *Oat1* KO microbiomes, they were not significantly enriched. However, 3-hydroxybutyrate dehydrogenase [EC: 1.1.1.30], which catalyzes the interconversion of acetoacetate to 3-hydroxybutyrate, was significantly elevated in the knockout microbiome (adj *p*-value = 6.7 × 10^−4^, Log2FC = 6.1) (Figure 3). Thus, while the functional predictions indicate butyrate production is not significantly enriched in the *Oat1* KO, which is consistent with the data in Figure 1 (i.e., shrinking of butyrate-producing phyla *Firmicutes* in the *Oat1* KO), pathways leading to 3-hydroxybutyrate, a known in vitro OAT substrate that is elevated in the *Oat1* KO mouse, are significantly enriched [35]. Importantly (for the ensuing discussion), 3-hydroxybutyrate is a suspected HDAC inhibitor [36,37].

### 2.4. The Oat1KO Microbiome Favors Fatty Acid Synthesis, Including the Oat1-Interacting Medium-Chain Fatty Acid (MCFA) Heptanoate (C7) and Odd-Chain-Length Fatty Acids

Our previous analyses suggested fatty acid metabolism is altered in the *Oat1* KO microbiome [1], though it was not explored. However, as shown in Table 2, OAT1 interacts with medium-chain fatty acids (C6–C8), which are key signaling molecules that have high OAT1 affinity [21]. Thus, we sought to determine whether fatty acid synthesis was elevated in the *Oat1* KO mouse using our DESEQ2-based analysis.

**Figure 4:**
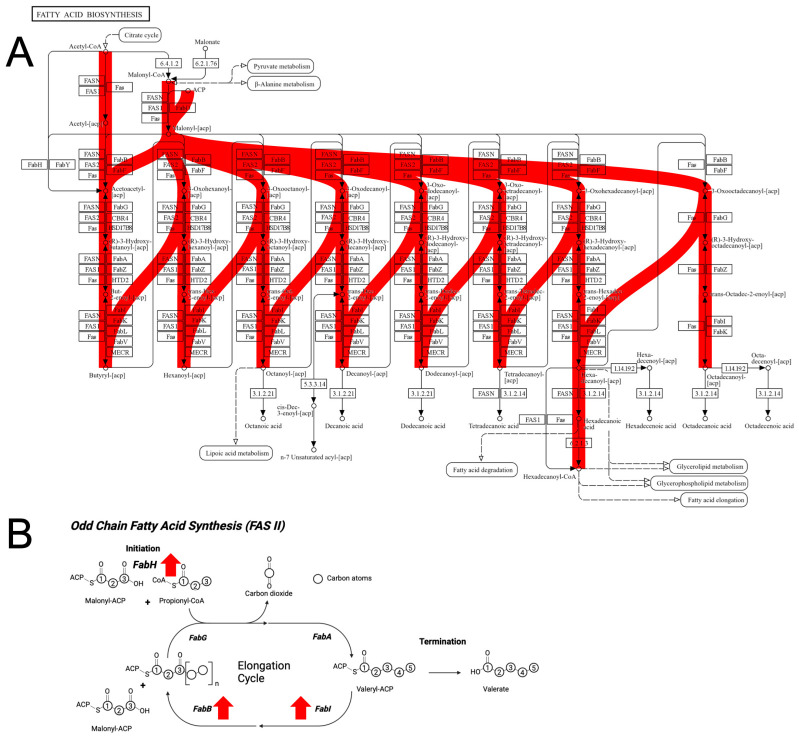
Pathway diagram describing medium- (C6-C12) to long-chain (C12+) fatty acid synthesis enrichment in the *Oat1* KO microbiomes. (**A**) Fatty acid elongation is enriched in the *Oat1* KO due to increases in bacterial Fatty acid synthase, bacteria type [EC:2.3.1.-] (*fas*) (Log2 fold-change 3.25, adj. *p*-value = 0.0096). Red-highlighted enzymatic steps indicate a significant increase in the abundance of the enzyme in the *Oat1* KO microbiome. (**B**) Diagram explaining odd-chain fatty acid synthesis. Propionyl-CoA, and thus propionate, are rate-limiting in odd-chain fatty acid synthesis and can be used by *fabH* instead of acetyl-CoA. Red arrows indicate enzymes that are significantly elevated in the *Oat1* KO (e.g., *fabH*, *fabB*, and *fabI*). Heptanoate production appears elevated in the *Oat1* KO microbiomes according to the odd-chain fatty acid synthesis pathway present in prokaryotes. As indicated in Table 2, heptanoate (Ki 17 µM) interacts with OAT1 with high affinity.

The results of the differential abundance analysis were mapped onto the KEGG pathway: ko00061 (fatty acid biosynthesis) (Figure 4A,B). We were able to reconstruct fatty acid elongation due to a signifcant increase in the enzyme complex: *fatty acid synthase*, *bacteria type* (Log2FC = 3.25, adj. *p*-value = 0.009). In fatty acid synthesis, propionate may be used instead of acetyl-CoA to produce odd-chain-length fatty acids, a pathway exclusive to prokaryotes [44,45]. Propionyl-CoA is a major limiting factor in odd-chain fatty acid biosythesis, so the significant elevations of propionate synthesis found earlier led us to hypothesize that odd-chain-length fatty acid biosynthesis may also be enriched in the *Oat1* KO.

In our further analyses, we were able to identify significant increases in individual bacterial fatty acid biosynthesis enzymes: *FabH*, and *FabI*. Other members of the *Fab* group were elevated, but did not reach statistical significance (adj. *p*-value > 0.05). *FabH* is able to initiate fatty acid synthesis using propionyl-CoA instead of acetyl-CoA to produce odd-chain fatty acids of chain length (C5) or greater (Figure 4B). Furthermore, fatty acid synthase, bacteria type, which participates in type II fatty acid synthesis (FAS II) is able to elongate propionyl-CoA into odd-chain-length medium- and long-chain fatty acids, which are transported by OAT1 [22].

Taken together, there appears to be an increase in fatty acid biosynthesis and elongation, which may utilize elevated propionate levels to synthesize odd-chain-fatty acids, including the signaling medium-chain fatty acids, which interact with OAT1, such as heptanoate (Ki 17 µM) (Table 2).

### 2.5. Serum Propionate Derivatives Increase Under Diminished OAT1 Function and Are Dependent on the Gut Microbiome

We sought to investigate whether the changes in propionate synthesis in the gut microbiome had an impact on serum levels of SCFAs when *Oat1* was deleted in mice. Propionyl-glycine, a propionate derivative and an indicator of propionyl-CoA overflow, was significantly increased in the *Oat1* KO mice [38,39,40]. This result led us to reevaluate data in an earlier study on the role of OAT1 in systemic lipid metabolism [22]. In the supplementary data for that study, propionyl glycine was elevated 6.6-fold (*n* = 5, *p*-adj = 0.006) in *Oat1* KO mice. In contrast, this was not seen in mice lacking the closely related organic anion transporter *Oat3* (Slc22a8), where there were minimal changes in gut microbiome pathways regulating propionate synthesis. For example, propionate CoA-transferase [EC:2.8.3.1] was only slightly elevated in the *Oat3* KO microbiomes (Log2FC = 1.44, *p*-adj = 0.017).

**Figure 5:**
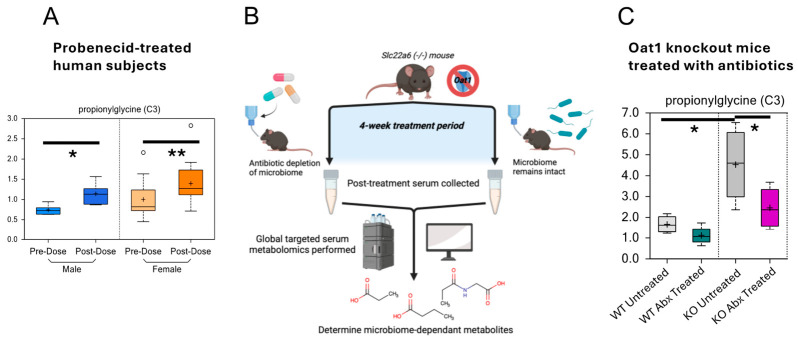
Serum propionate derivatives are (1) elevated in knockout mice and humans treated with probenecid and (2) sensitive to antibiotics in a gut microbiome depletion experiment. (**A**) Boxplots showing elevated levels of propionyl-glycine from previous data on 20 human subjects treated with the OAT1-inhibiting drug probenecid [47]. While the *Oat1* knockout has no OAT1 function due to gene disruption, the drug probenecid is used experimentally and clinically to block OAT1 function at the level of the transporter protein. Probenecid acts as a competitive inhibitor of metabolite transport. (**B**) Illustration of the treatment regimen and subsequent metabolomics analysis of *Oat1* KO and wildtype mice treated with broad-spectrum antibiotics (Abx) as previously described [2]. (**C**) Boxplots of absolute levels of propionyl-glycine measured in Abx-treated wildtype, untreated wildtype, knockout untreated, and knockout Abx untreated mice (*n* = 4 each). Untreated *Oat1* KO mice had much higher propionyl-glycine levels than their WT counterparts. However, antibiotic treatment of the *Oat1* KO significantly decreased serum propionyl-glycine levels, suggesting gut-microbiome dependence of propionyl-glycine level in the absence of OAT1 function. Of note, there was no elevation of propionyl-glycine in the Oat3 KO and only minimal change in bacterial propionate synthesis. Circles on boxplots represent outliers. (*) *q*-value < 0.05, (**) *q*-value < 0.01.

Since propionyl-glycine has been shown to be important in humans [46], we reanalyzed metabolomics from human subjects previously treated with probenecid, a well-known OAT1 inhibitor [47]. Probenecid is widely used both experimentally and clinically [2,3,5]. It functionally blocks renal OAT-mediated transport at the level of the transporter protein in vivo and in vitro as measured by OAT1 transport assays in transfected cells [35]. Five hours after probenecid was administered, there was a significant elevation of serum propionyl-glycine levels (Figure 5A). As in the knockout mice, this indicated elevated levels of propionyl-glycine are likely a result of diminished OAT1 function.

We sought to investigate whether elevated propionyl-glycine levels were microbiome-dependent. We therefore reanalyzed previous data from mice treated with an antibiotic cocktail as a means to deplete the microbiome (Figure 5B,C) [1,2]. Serum metabolomics also revealed that the antibiotic depletion of the microbiome in *Oat1* KO mice significantly reduced levels of propionyl-glycine, suggesting that the gut microbiome is largely responsible for the serum elevations observed in the *Oat1* KO mouse. Again, it is worth noting that propionyl-glycine is an end product of propionate metabolism and is thus an indicator of propionate levels [46,48,49]. In addition, we have previously reported 3-hydroxypropionate to be elevated in the *Oat1* KO mice [32]. While the pathways involving this propionate metabolite are less well-characterized, this Oat1-dependent metabolite also appears to arise from the microbiome and likely indicates flux through the propionate pathway.

Together, this provides strong evidence that SCFAs are elevated in the *Oat1* KO mice, where the gene is disrupted, and also in humans when the OAT1 transporter is functionally blocked by probenecid. The analysis also shows that the SCFAs originate in the gut microbiome of the *Oat1* KO mouse, which is consistent with strong enrichment at all steps of the gut bacterial propionate pathway in the *Oat1* KO mice (Figure 3).

In addition, we applied a similar approach to 3-hydroxybutyrate, a known OAT1 substrate that is elevated in the knockout mice. In an analysis of female *Oat1* knockout mice, the elevation of 3-hydroxybutyrate was suppressed through gut microbiome depletion with antibiotics according to a previously published protocol [2]. This indicates that, as with propionyl-glycine, the elevation of 3-hydroxybutyrate in the absence of OAT1 is dependent on the gut microbiome. Furthermore, in a reanalysis of our data from humans treated with probenecid [47], 3-hydryoxybutyrate was found to be elevated in the plasma and decreased in the urine. Like propionate and butyrate, 3-hydroxybutyrate is a suspected HDAC inhibitor; however, its effects may be context-dependent [36,50].

## 3. Discussion

Previous studies have indicated that the gut microbiome acts to restore homeostasis when transporter-mediated tubular secretion is diminished [1]. For example, the loss of OAT1 leads to an increase in systemic uric acid levels, which the gut microbiome compensates for by reducing its own production of urate [1]. While uric acid may have some beneficial properties as a potent antioxidant, in high concentrations, it can cause redox stress and is deleterious to both the gut microbiome and host organism, resulting in metabolic diseases such as gout and cardiovascular disease [51,52]. The toxic effects of high uric acid may explain why the gut microbiome decreases its own production in the *Oat1* KO. Consistent with the RSST, this is an example of the compensatory role the gut microbiome may have when transporter-mediated tubular secretion is diminished. This type of mechanism may occur alongside increased intestinal excretion of urate by ABCG2 as renal function declines [53,54].

**Figure 6:**
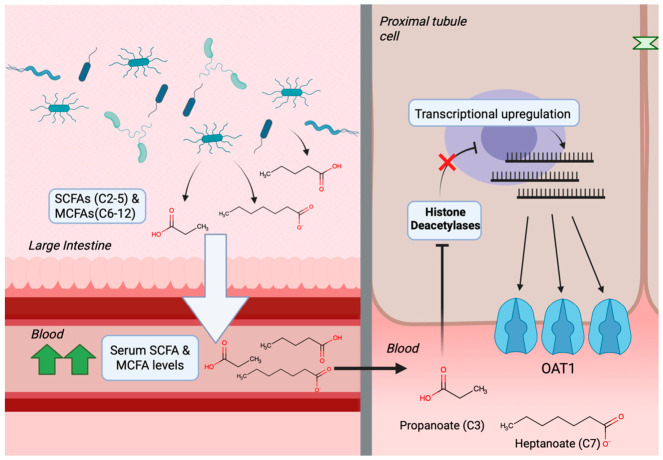
Schematic of gut microbiome-mediated regulation of OAT1 expression. Anaerobes produce short-chain fatty acids (SCFAs) and medium-chain fatty acids (MCFAs) which are absorbed into the blood by enterocytes [30,38,44,55,56,57]. SCFAs and MCFAs inhibit histone deacetylases, which affect OAT expression [19,38]. SCFAs and MCFAs are transported into proximal tubule cells through OAT1 and other pathways. SCFAs and MCFAs then induce OAT-dependent secretion in the kidney [19]. Ultimately, when OAT1 is deleted, there is a marked increase in gut microbiome SCFA and MCFA production to restore OAT1-mediated tubular secretion of fatty acids.

Here we have shown a gut-microbiome-mediated compensatory effect involving medium-chain and short-chain fatty acids to restore OAT-mediated tubular secretion (Figure 6). Previous in vitro studies have indicated that certain SCFAs (C2–C5) and MCFAs (C6–C12) are transported by OATs [20,21,22,48]. For example, heptanoate (C7) has been previously shown to be transported by OAT1 (Ki 16.7) and inhibits histone deacetylases (HDACs) within the cell [21,38]. This regulatory effect can be seen with other SCFAs and MFCAs as well. A previous study by others has indicated that OAT1 and OAT3 expression in vitro are significantly upregulated (3-fold increase) when epithelial tubules are exposed to the SCFA propionate, aiding the secretion of uremic toxins such as indoxyl sulfate [19]. This result was interpreted by the researchers in terms of the RSST. These findings, in combination with the results of our study, indicate a homeostatic role that the microbiome plays in restoring tubular secretion when loss of OAT-function (i.e., transport of signaling fatty acids) is ‘sensed’. This feedback effect is consistent with the Remote Sensing and Signaling Theory and, along with urate, is another example of gut-microbe and kidney crosstalk mediated by OAT1.

The results of our metagenomic sequencing of the *Oat1* KO microbiomes indicate a marked increase in propionate synthesis via succinate- and 1,2-propanediol-dependent pathways in the bacteria (Figure 3). These findings were supported by major taxonomical changes in the *Oat1* KO favoring propionate-producing phyla *Bacteroidota*. Additionally, metagenomic sequencing revealed increased fatty acid elongation through the bacterial pathway fatty acid synthesis II (FAS II) (Figure 4). Given the increase in both propionate synthesis and fatty acid elongation, our results indicate an elevation in odd-chain fatty acid synthesis in the *Oat1* KO bacteria, including MCFAs such as heptanoate (C7) and long chain fatty acids such as pentadecanoate (C15). Pentadecanoic acid also appears to inhibit histone deacetylase and is elevated in the *Oat1* KO [22,38]. It is worth emphasizing that many of the MCFAs, SCFAs, and their derivatives directly interact with one or more OAT-subfamily transporters in in vitro transport assays [20,21].

Serum metabolomics was used to investigate whether this change in bacterial propionate synthesis could be detected systemically in the *Oat1* KO mouse. Elevated propionyl-glycine, a marker for propionyl-COA overflow [46], was detected in the *Oat1* KO serum, indicating greater propionate abundance in the serum of the *Oat1* KO mouse. Furthermore, this effect was observed in a reanalysis of our data from human subjects treated with the OAT1-inhibiting drug probenecid, who had a similar increase in propionyl-glycine following treatment (Figure 5) [41]. Additionally, odd-chained fatty acids are degraded into propionate by the host [44,46]. Thus, elevated propionate derivatives may result from greater odd-chain fatty acid oxidation as well [46].

Finally, in order to validate propionyl-glycine was of microbial origin, we reanalyzed the serum metabolomics from a previous study of *Oat1* KO mice with an antibiotic-induced depletion of the gut microbiome and found significant decreases in propionyl-glycine (Figure 5) [2]. Overall, these results indicate a functional shift in the gut microbiome of the *Oat1* KO mouse leads to elevated systemic concentrations of propionate-derivatives in the serum. Taken together with previous findings, our findings support a homeostatic model in which the microbiome responds to the loss of OAT1 function via SCFA and MCFA production (Figure 6).

Furthermore, we analyzed 3-hydroxybutyrate, an OAT1 substrate, which is elevated in *Oat1* knockout mice [21]. We did this because 3-hydroxybutyrate is a suspected HDAC inhibitor [36]. The increase of 3-hydroxybutyrate in the absence of OAT1 was dependent on the gut microbiome [2]. In humans treated with probenecid, 3-hydryoxybutyrate was increased in the serum with a decrease in the urine. This is, effectively, a hallmark of OAT-dependent tubular secretion.

Considered together, our data indicates a compensatory feedback loop, which comes into play when tubular secretion of SCFAs, MCFAs, and 3-hydroxybutyrate is diminished. In an attempt to restore tubular secretion by OAT1 (and possibly other organic anion transporters), the gut microbiome increases its production of propionate, MCFAs and odd-chain fatty acids, which inhibit HDACs that suppress OAT expression [19,38,58]. However, because the *Oat1* gene is disrupted, it is not possible to restore homeostasis, and this leads to a chronic reset of the gut microbiome in the *Oat1* KO mouse [5,6,7,8,9,10,11].

It is important to note that chronic kidney disease (CKD) is also characterized by a progressive loss of kidney function defined in part by the loss of tubular secretion via OATs [59]. Metagenomic studies performed on CKD patients have revealed that the gut microbiome experiences a functional shift away from short-chain fatty acid production, and thus a potential loss of SCFA-mediated OAT upregulation [60,61]. Our results indicate that, in the setting of OAT1 loss, increased SCFA and MCFA production by the gut microbiome is an important homeostatic response to restore tubular function in the kidney.

When the data is interpreted in the context of in vitro studies by others [19], an in vivo feedback loop connecting gut microbial-derived SCFAs and MCFAs to renal proximal tubule uptake by OAT1 becomes clear (Figure 6). This bidirectional pathway is very compatible with the Remote Sensing and Signaling Theory, which emphasizes the key roles of SLC and ABC multispecific transporters in inter-organ and inter-organismal remote communication. In this case, SCFAs and MCFAs are small molecules with high informational content acting upon HDACs to change the chromatin state such that OAT1 expression is modulated.

## 4. Materials and Methods

### 4.1. Functional Metagenomics

Seven *Oat1* KO and 8 wildtype C57/BL6 mice aged 2–3 months were used in this study. *Oat1* KO mice were generated as described previously [35]. Samples for fecal metagenomics were collected from these mice in sterile microfuge tubes, snap frozen, and then stored at −80 °C until sent off for sequencing. The UCSD microbiome core handled the nucleotide extraction, library prep, and metagenomic sequencing of the microbiomes. Reads were uploaded to One Codex, a metagenomic read manager, and taxonomies were assigned using the Transnetyx (Cordova, TN, USA) database.

Read quality was first assessed using FASTQC (v0.12.1) [62]. Murine host reads were removed with bowtie2 (v2.5.4), utilizing the GRCm39 as a mouse reference for C57BL6 [63]. Sequencing adapters were subsequently trimmed using Trimmomatic (v0.39) [64], and reads <70 bp were removed. These trimmed reads were then assembled into contigs using metaSPADES (v 3.13.1) [65], an assembler tailored for metagenomic reads. Coding sequences were assigned to these contigs using Prodigal (v 2.6.3) [66] and annotated using Kofam Scan (v 1.3.0) [67]. High-confidence annotations were reported by Kofam Scan, which were calculated according to sequence length and low E-value. These were used for subsequent analyses. MMseqs2 (v 13.45111) [68] was used to cluster coding sequences to form a non-redundant database of genes. CoverM (v 0.6.1) [69] was used to align the original reads to this database to create a count table. Afterwards, any entries sharing the same KEGG ID were then collapsed into one entry. The count table generated was then used for differential abundance analysis of bacterial genes.

### 4.2. Reanalysis of Metabolomics Datasets

We reanalyzed metabolomics data from our previous studies, focusing on a subset of metabolites surrounding propionate metabolism in *Oat1* KO mice, antibiotic treated mice, and humans treated with probenecid [2,47]. The methods for the metabolomics are described in these studies. Briefly, global targeted metabolomics was performed by Metabolon Inc. (Morrisville, NC, USA). Human and mouse serum were subjected to ultra-high-performance liquid chromatography–tandem mass spectrometry (UHPLC-MS/MS). For convenience, we briefly describe these previous studies below. Please refer to the original studies for more details.

### 4.3. Human Probenecid Study

As previously described [47], blood and urine were collected from 20 healthy human participants (14 women and 6 men) before and 5 h after an oral dose of 1 g of probenecid. Global targeted metabolomics was performed on the collected samples by Metabolon Inc. and pre- and post-treatment comparisons were made. In total, 1234 unique metabolites were measured in the participants. Please refer to original study for more details.

### 4.4. Antibiotic Depletion Experiment

As described in our previous study [2], 8 *Oat1* KO and 8 wildtype C57/BL6 mice were divided into antibiotic-treated and untreated groups (*n* = 4, each). The mice were given a 125 mL antibiotic cocktail in place of water consisting of 1 mg/mL of neomycin sulfate 1 mg/mL of ampicillin, 1 mg/mL of metronidazole, and 0.5 mg/mL of vancomycin. The antibiotic cocktails were replenished every other day. The untreated mice were given a vehicle control. After a 4-week treatment period, serum metabolomics was performed by Metabolon Inc. (Morrisville, NC, USA). Comparisons between treated and untreated, as well as *Oat1* KO versus wildtype metabolomics, were made. To confirm a broad depletion of the gut microbiome in the treated mice, 16S amplicon sequencing was performed. Please refer to the original study for details on the metabolomics. 

### 4.5. Statistics

A differential abundance analysis of bacterial genes was accomplished using DESEQ2 (v1.4.2.1) [70]. DESEQ2 performs a multiple comparisons correction when calculating differential abundance; therefore, genes with an adjusted *p*-value (adj. *p* < 0.05) were deemed statistically significant. For the metabolomics reanalyzed in this study, *p*-value and False Discovery Rate (FDR) were calculated using Welch’s two-sample *t* test. Compounds with an *q*-value < 0.05 were deemed statistically significant.

### 4.6. Visualization

Ggplot2, a data visualization package in R, was used for most of the figures generated here. ggKegg (v1.0.13), an R package that uses KEGG API to create pathway visualizations, was used to generate the pathway diagrams.

## 5. Conclusions

The in vivo results presented here indicate bidirectional OAT1-mediated interactions between the kidney and gut microbiome via small molecules. In the context of the Remote Sensing and Signaling Theory, there are now three such RSST bidirectional feedback loops between renal OAT1 and microbiome-derived small molecules participating in key homeostatic pathways in the body. These pathways surround: (1) indoxyl sulfate; (2) uric acid; and (3) SCFAs and MCFAs. Importantly, key points of regulation of these three pathways are partly or largely distinct: (1) AHR for indoxyl sulfate; (2) net gut microbial urate production and the activity of other renal proximal tubule transporters, as well as intestinal ABCG2 (in CKD) for uric acid; and (3) chromatin modification via HDACs for SCFAs and MCFAs [1,5,11,19,23,56,71,72]. Thus, these three seemingly “canonical” RSST loops, while overlapping, have relatively distinct regulatory points. Whether these three RSST loops are constituents of a broader “meta-loop” centered around OAT1 remains to be determined. Recent articles on CKD, especially in the context of protein-bound uremic toxins, have emphasized the RSST as a conceptual framework [6,10,16]. It is likely that future translational and clinical studies, which can be interpreted within this framework, will add new insights into the pathophysiological implications of RSST, and, potentially, help set the stage for better CKD outcomes as suggested by several groups [10,16]. In the future, organ-on-a-chip studies seem likely to add new insights into RSST loops [73].

## Figures and Tables

**Table 1:** Bacterial genes involved in propionate synthesis elevated in the *Oat1* KO.

	Log2FC	Adj. *p*-Value
propionyl-CoA synthetase [EC:6.2.1.17]	9.72	1.11 × 10^−9^
propionaldehyde dehydrogenase [EC:1.2.1.87]	3.58	1.2 × 10^−4^
propanediol dehydratase large subunit [EC:4.2.1.28]	4.39	5.46 × 10^−6^
propanediol dehydratase medium subunit [EC:4.2.1.28]	4.61	0.0019
lactaldehyde reductase [EC:1.1.1.77]	3.24	9.18 × 10^−31^
glycerol dehydrogenase [EC:1.1.1.6]	4.19	0.035
alcohol dehydrogenase, propanol-preferring [EC:1.1.1.1]	8.76	5.71 × 10^−15^

**Table 2:** Short and medium-chain fatty acids and their interactions with OAT1 and OAT6.

Organic Anions	OAT1 (Ki µM) [Reference]	OAT6 (Ki µM) [Reference]	HDAC Inhibition [38,39,40,41]
C2 (Acetate)	-	-	✓
C3 (Propionate)	8180 [20]	279 [20]	✓
C4 (Butyrate)	3500 [21]	82 [21]	✓
3-Hydroxybutyrate	3220 [21]	191 [21]	✓
C5 (Valerate)	-	-	✓
C6 (Hexanoate)	38 [21]	9 [21]	✓
C7 (Heptanoate)	16.7 [21]	8.2 [21]	✓
C8 (Octanoate)	5.41 [42]	-	
Succinate	4825 [43]	-	

## Data Availability

Sequencing data were uploaded to the Sequence Read Archive (SRA) and are available under the accession no. PRJNA1012006.

## Abbreviations

RSST	Remote Sensing and Signaling Theory
OAT	Organic anion transporter
SCFA	Short-Chain Fatty Acid
MCFA	Medium-Chain Fatty Acid
WT	Wildtype
KO	Knockout
KEGG	Kyoto Encyclopedia of Genes and Genomes
HDACs	Histone Deacetylase
CKD	Chronic Kidney Disease
CDS	Protein Coding Sequence
CPM	Counts Per Million
EGF	Epidermal Growth Factor
GPCRs	G-protein-Coupled Receptors
AUC	Area Under the Curve
FAS II	Fatty Acid Synthesis, Type II
AHR	Aryl Hydrocarbon Receptor
